# Strategies to improve lung absorption of intratracheally administered compounds in Drug Discovery

**DOI:** 10.1186/1476-9255-10-S1-P35

**Published:** 2013-08-14

**Authors:** Elena Calama, Montserrat Vives, Isabel Ramis, Sonia Espinosa, Juan Pérez, Josep Maria Huerta, Jorge De Alba, Peter Eichhorn

**Affiliations:** 1 Respiratory Therapeutic Area Discovery, Almirall, R&D Centre, 08980 Barcelona, Spain; 2 Pharmacokinetics and Drug Metabolism, Almirall, R&D Centre, 08980 Barcelona, Spain; 3 Structural Analysis, Almirall, R&D Centre, 08980 Barcelona, Spain; 4 Galenic Development, Almirall, R&D Centre, 08980 Barcelona, Spain

## 

The key factors governing the absorption of drugs from the lungs are deposition pattern, dissolution rate, solubility and permeability. Whereas the thin epithelium in the respiratory airways is generally considered to constitute a minor barrier in the overall absorption process, the other two components depend, among other parameters, on the formulation and the particle size. This is of particular importance for compounds with very low solubility.

The aim of this work was to evaluate strategies to improve the rate and extent of lung absorption in rats which were administered with suspensions of low-solubility compound by the intratracheal route. The two approaches evaluated were (a) the reduction of the particle size by micronization to increase the total surface area and hence to speed up the dissolution, and (b) the addition of methylcellulose to the vehicle, thereby enhancing its viscosity and potentially increasing the compound solubility.

For the pharmacokinetic studies performed with male Wistar rats, the test compounds were formulated in PBS (pH 7.4)/0.2 % Tween-80 with or without the addition of 0.3 % methylcellulose (Sigma-Aldrich M0512, 4000 cPs). Compounds were used either directly as obtained from the chemical synthesis or after a micronization process. Suspensions were delivered into the respiratory tract of the rats using a Penn-Century liquid microsprayer (200 uL). Disappearance of the compounds from the target organ within a 24-h period post-dose was monitored by UPLC-ESI-MS/MS.

The pharmacokinetic data obtained for chemically diverse compounds indicated that both strategies affected in a very distinct, compound-dependent fashion the pulmonary absorption. In conclusion, micronization and, if this is not technically feasible, addition of methylcellulose to the vehicle may constitute a suitable means of enhancing the pulmonary absorption of compounds delivered into airways of rats. Based on the presented findings, predicting the effect of either methodology appears to be difficult and thus requires a case-by-case evaluation.

